# Medical-grade polycaprolactone scaffolds made by melt electrospinning writing for oral bone regeneration – a pilot study in vitro

**DOI:** 10.1186/s12903-019-0717-5

**Published:** 2019-02-01

**Authors:** A. Fuchs, A. Youssef, A. Seher, G. Hochleitner, P. D. Dalton, S. Hartmann, R. C. Brands, U. D. A. Müller-Richter, C. Linz

**Affiliations:** 1 Department of Oral and Maxillofacial Plastic Surgery, University Hospital Würzburg, Pleicherwall 2, 97080 Würzburg, Germany; 2 Department for Functional Materials in Medicine and Dentistry, University Hospital Würzburg, Pleicherwall 2, 97080 Würzburg, Germany; 3 Interdisciplinary Center for Clinical Research, University Hospital Würzburg, Josef-Schneider-Straße 2, 97070 Würzburg, Germany

**Keywords:** Melt electrospinning writing, Polycaprolactone, Scaffold, Guided bone regeneration

## Abstract

**Background:**

The spectrum of indications for the use of membranes and scaffolds in the field of oral and maxillofacial surgery includes, amongst others, guided bone regeneration (GBR). Currently available membrane systems face certain disadvantages such as difficult clinical handling, inconsistent degradation, undirected cell growth and a lack of stability that often complicate their application. Therefore, new membranes which can overcome these issues are of great interest in this field.

**Methods:**

In this pilot study, we investigated polycaprolactone (PCL) scaffolds intended to enhance oral wound healing by means of melt electrospinning writing (MEW), which allowed for three-dimensional (3D) printing of micron scale fibers and very exact fiber placement. A singular set of box-shaped scaffolds of different sizes consisting of medical-grade PCL was examined and the scaffolds’ morphology was evaluated via scanning electron microscopy (SEM). Each prototype sample with box sizes of 225 μm, 300 μm, 375 μm, 450 μm and 500 μm was assessed for cytotoxicity and cell growth by seeding each scaffold with human osteoblast-like cell line MG63.

**Results:**

All scaffolds demonstrated good cytocompatibility according to cell viability, protein concentration, and cell number. SEM analysis revealed an exact fiber placement of the MEW scaffolds and the growth of viable MG63 cells on them. For the examined box-shaped scaffolds with pore sizes between 225 μm and 500 μm, a preferred box size for initial osteoblast attachment could not be found.

**Conclusions:**

These well-defined 3D scaffolds consisting of medical-grade materials optimized for cell attachment and cell growth hold the key to a promising new approach in GBR in oral and maxillofacial surgery.

## Background

In the field of oral and maxillofacial surgery, membranes are used for a broad spectrum of indications including Guided bone regeneration (GBR) applications. In this context, membranes function as a barrier between fast-proliferating soft tissues, such as fibrous connective tissue or epithelium and the rather slow-proliferating bone [1]. Using scaffold geometries and surfaces that are tailored to the requirements of bone tissue may promote bone regeneration in GBR.

In general, membranes that are currently used for maxillofacial applications, such as GBR, can be broadly divided into resorbable and non-resorbable categories. Membranes of the latter category offer good biocompatibility and high mechanical stability. Thus they suit very well as placeholders and barriers in GBR. On the other hand, non-resorbable membranes require a second operation for their removal, pose a risk of mucosal perforations due to their high level of stiffness and therefor go along with higher morbidity, increased costs and increased expenditure of time. By contrast, resorbable membranes currently mostly consist of collagen, synthetic aliphatic polyesters, or their co-polymers [2–4]. Normally, resorbable membranes that are currently used show excellent biocompatibility, a reduced risk of wound dehiscence, and good biodegradability. On the other hand, especially for in GBR most commonly used collagen membranes, a rapid loss of mechanical stability is apparent, and their clinical handling is often not ideal due to their low resilience and lubricity. Furthermore, as these membranes are either of allogeneic or xenogeneic origin, a potential risk of transmission of infection as well as potential legal, ethical or religious limitations have to be considered [2, 5]. In total, all available membrane systems for oral applications maintain certain drawbacks. One promising approach to producing membranes/scaffolds that compensate for the disadvantages of currently available membranes is electrospinning [6] - and even more recently, melt electrospinning writing (MEW) [7]. Electrospinning is a versatile and easy technique to produce scaffolds for biomedical applications. In electrospinning, an electrically charged, viscous polymer jet is ejected from a spinneret and “drawn” through the air in the direction of a collector with opposite electrical potential where the fibers form either chaotic mats or well-defined structures depending on which electrospinning method is being used [8]. Regarding the initial state of the polymer, two different types of electrospinning can be distinguished: solution electrospinning and MEW. In solution electrospinning, polymers are dissolved in organic solvents, such as chloroform or dimethylformamide, which evaporate when the polymer jet is ejected towards the collector. Disadvantages of the solution spinning process include the resulting solvent residues in the fibers as well as the fact that only uncontrolled fiber deposition is feasible due to electrostatic forces and concomitant increased bending and deflection of the polymer jet [9, 10]. MEW, in contrast allows for a very exact placement of fibers made from medical-grade polymers up to sub-micrometer scale without the use of any solvents and with no risk of residual toxic solvents in the finished scaffold [11–15]. This placement can be achieved because the polymer is not dissolved, but melted. Process parameters allow for a controlled fiber placement, which makes the computer-aided design- and manufacturing (CAD/CAM) of 3D scaffolds feasible. Cell growth on scaffolds can thus be optimized by varying pore sizes and interconnectivity.

For this study, medical-grade PCL scaffolds with box-shaped pores with sizes of 225, 300, 375, 450, and 500 μm were fabricated for a perspective application in GBR, seeded with an osteoblast-like cell line, and evaluated to determine which pore size best promotes cell growth.

## Methods

### Materials

All scaffolds that were used in this pilot study consisted of medical-grade PCL purchased from Corbion Inc. (Gorinchem, Netherlands, PURASORB PC 12). It was divided into 50 mL falcon tubes in an argon atmosphere and stored at − 80 °C until used.

### Fabrication via MEW

**Fig. 1:**
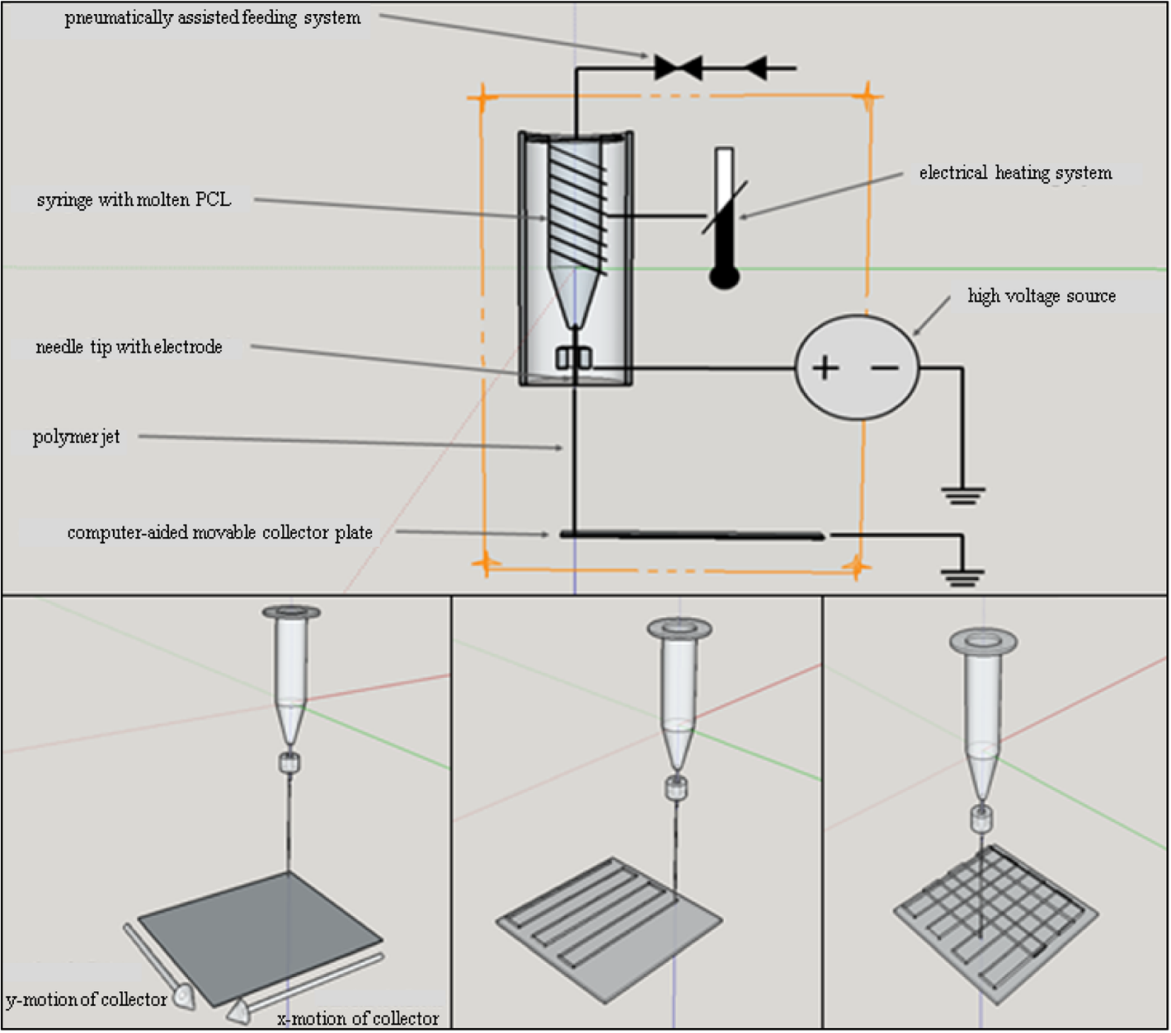
Design of MEW device and schematic of the MEW process of the scaffolds

For scaffold fabrication, we used a custom-made MEW device that was designed and built by the Department for Functional Materials in Medicine and Dentistry Würzburg University Hospital (Fig. 1) [11]. The machine parameters were adjusted to produce fibers with diameters of approximately 20 μm. The PCL was heated inside the device at 73.0 °C ± 1.0 °C in a disposable plastic syringe with a 22G needle attached to it (both from Nordson EFD Deutschland GmbH, Pforzheim, Germany). A pneumatic pressure of 1.2 bars was applied to the melted polymer, and an acceleration voltage of 6 kV was used between the tip of the needle and the stainless-steel collector. The distance between both was 4 mm, and the collector moved at a rate of 400 mm/min. MEW was performed at an ambient temperature of 21.4 °C ± 0.4 °C and a humidity level of 38.5 ± 3.5%. The polymer was used within 5 days.

### Scaffold designs

One series of box-structured MEW scaffolds was evaluated for all experiments. MEW was performed by alternating the layer deposition via 0° and 90° layers (15 × 15 mm^2^) in each direction with turning loops. Five layers were deposited in each direction (0° and 90°) such that 10 fibers overlapped at intersections. Scaffolds with fiber diameters of 20 μm and different filament spacings of 225 μm, 300 μm, 375 μm, 450 μm and 500 μm were fabricated in this manner. 100% ethanol was used to separate the scaffolds from the metal collector.

### Scanning electron microscopy (SEM)

All different scaffolds were characterized by SEM imaging to evaluate the accuracy of the MEW process. MEW scaffolds were washed four times with Phosphate Buffered Saline (PBS, Invitrogen, Karlsruhe, Germany). After fixation with 6% glutaraldehyde (Merck, Darmstadt, Germany), the scaffolds were dehydrated in rising concentrations of ethanol and finally dried with hexamethyldisilazane (Thermo Fisher Scientific, Waltham, USA) and sputter coated with platinum using a Leica EM ACE600 (Leica Microsystems GmbH, Wetzlar, Germany).

### Cell culture

Human osteoblast-like cell line MG63 was cultivated in Dulbecco’s Modified Eagle’s Medium (DMEM, Invitrogen, Karlsruhe, Germany), which was supplemented with 10% fetal calf serum (FCS, Invitrogen, Karlsruhe, Germany) and 1% penicillin and streptomycin (P/S, Invitrogen, Karlsruhe, Germany) in a prewarmed (37 °C) and humidified atmosphere with 5% CO_2_. The culture medium was changed every 3 days.

**Fig. 2:**
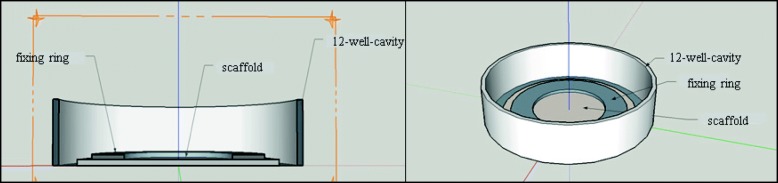
Schematic of cell culture experiments on the scaffolds

MG63 cells were seeded upon the box-structured scaffolds in an initial concentration of 3.0 × 10^5^ cells per scaffold. In vitro experiments were performed in 12-well multiwell plates (Thermo Fisher Scientific, Waltham, USA). Scaffolds were placed into a well and weighed down by a glass ring and glass beads to keep them from floating in the cell culture medium (Fig. 2). Positive controls consisted of cells with a glass ring and negative controls consisted of the cell culture medium and the cell culture medium with a glass ring and beads. Measurements for cell-viability, cell counting, and pH measurements were performed on days 2, 3 and 4 of cell settlement to assess initial cell attachment. Phalloidin staining, FDA/PI staining and the measurement of protein concentration were performed on day 4 of the cell culture.

### Cytocompatibility testing

#### Water-soluble tetrazolium (WST-1) assay

Cell viability reflects the amount of metabolically active cells and was tested with WST-1 assay (Roche Diagnostics, Mannheim, Germany). Prior to WST-1 testing, the fixation rings were removed, and the scaffolds were transferred to new 12-well multiwell plates. The scaffolds were then incubated with WST-1 reagent diluted 1:10 in medium for 30 min at 37 °C. 200 μl of the medium supernatant were then transferred into a cavity of a 96-well plate, and optical density was measured photometrically (Tecan Spektra Rainbow, Tecan, Crailsheim, Germany) at 440 nm.

#### Protein concentration assay

A quantitative assessment of the overall protein concentration of cells on scaffolds was performed according to the Lowry method [16] and the quantification of protein level was determined using a BSA standard curve. After washing the scaffolds three times with PBS (Invitrogen, Karlsruhe, Germany), they were covered with PBS and 20% NaOH (Merck, Darmstadt, Germany), sealed with MT-film (Tecan, Crailsheim, Germany), incubated at 80 °C for 1 hour, and finally neutralized with 20% HCl (Merck, Darmstadt, Germany). For the assessment of the protein concentration, DC Protein-Assay with reagent S, A and B was used (BIO-RAD, München, Germany). Standards and scaffold samples were added with working reagent (reagent A + S) and reagent B and stirred carefully. After 15 min, a color change to blue could be observed, and a photometric measurement at 750 nm was performed.

#### Cell counting

Prior to the counting process, cells were detached from the scaffold surface by incubation with 0.05% trypsin (Thermo Fisher Scientific, Waltham, USA) for 5 min and then washed and resuspended in DMEM. The cells that had settled upon the scaffolds were counted with a Coulter counter (Casy 1, Schärfe Systeme, Reutlingen, Germany).

#### pH-value measurement

pH values were determined prior to viability testing for the assessment of solubility and degradation activity of some PCL scaffolds by collecting supernatant from the cell culture medium and evaluating its pH values with a pH meter in combination with a pH electrode (SenTix 61, WTW, Weilheim, Germany).

#### Phalloidin staining

After removing the medium, the scaffolds were washed with PBS and then fixed with 4% paraformaldehyde (Histofix, Carl Roth, Karlsruhe, Germany) for 10 min. After rinsing the scaffolds with PBS, treating them with 0,1% triton x-100 (Sigma-Aldrich, Taufkirchen, Germany), and performing another PBS rinsing, 1% bovine serum albumin was applied for 30 min. Next, phalloidin (Thermo Fisher Scientific, Waltham, USA) was added, and the scaffolds were incubated for 1 h in complete darkness. Afterwards, the scaffolds were examined via fluorescence microscopy (BIOREVO BZ-9000, Keyence, Neu-Isenburg, Germany).

#### Fluorescindiacetate/Propidiumiodide (FDA/PI) staining

The scaffolds were washed once with PBS and then covered with a 1:9 mixture of FDA and PI (Sigma-Aldrich, Taufkirchen, Germany) for 10 s. After rinsing once more with PBS, the samples were immediately examined via fluorescence microscopy.

## Results

### Cytocompatibility

**Fig. 3:**
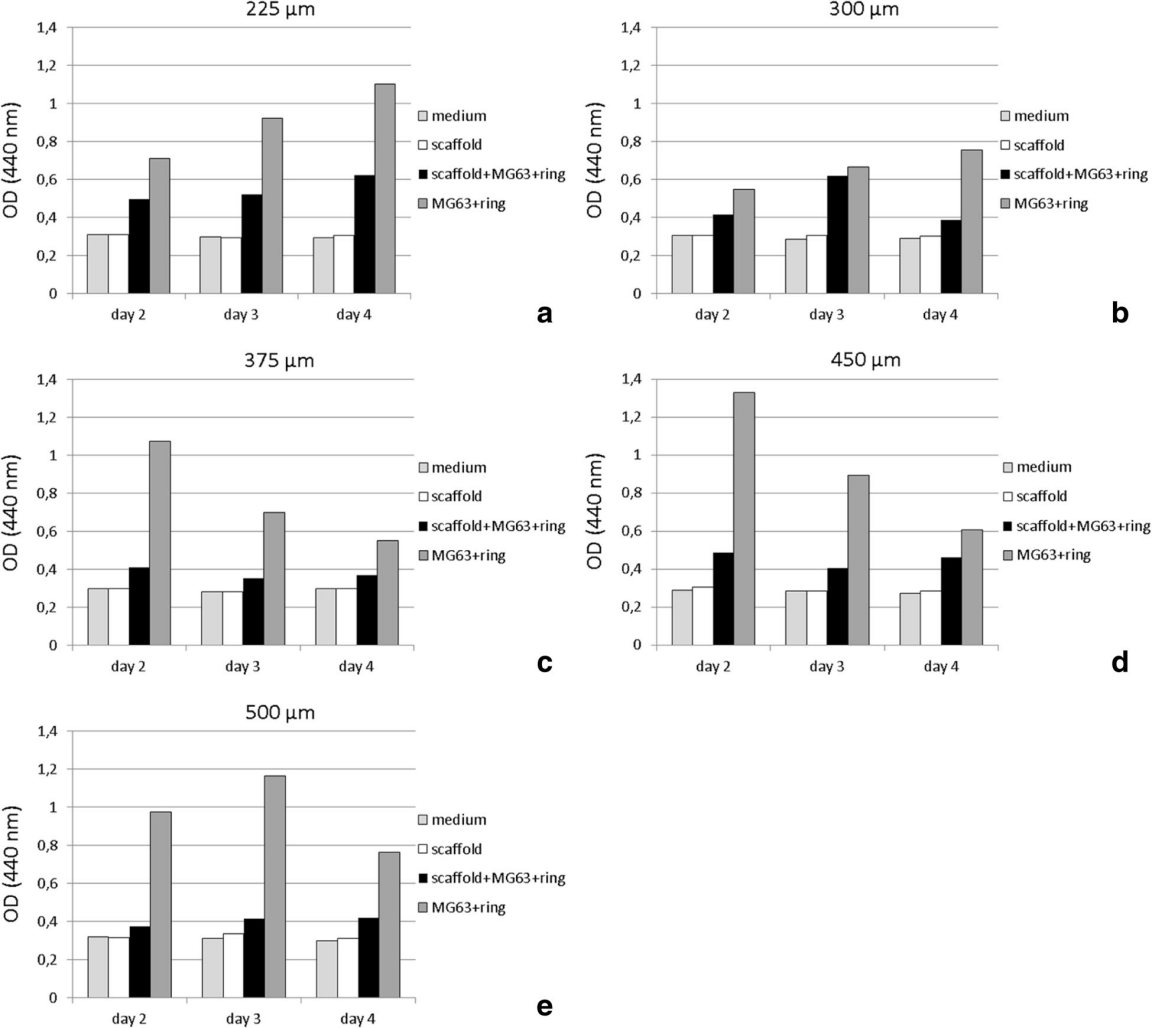
Cell viability of MG63 cells upon MEW box structured PCL scaffolds of different box sizes. Cell viability was measured via WST-1 after 2, 3, and 4 days. **a**) Box size 225 μm, **b**) box size 300 μm, **c**) box size 375 μm, **d**) box size 400 μm, **e**) box size 500 Μm*. medium*: pure DMEM, scaffold: MEW PCL scaffolds burdened with glass ring/beads, scaff+MG63 + ring: MG63 cells seeded on PCL scaffolds burdened with glass ring/beads, MG63 + ring: MG63 cells seeded on polystyrene wells of multiwell plates in presence of glass ring/beads

**Fig. 4:**
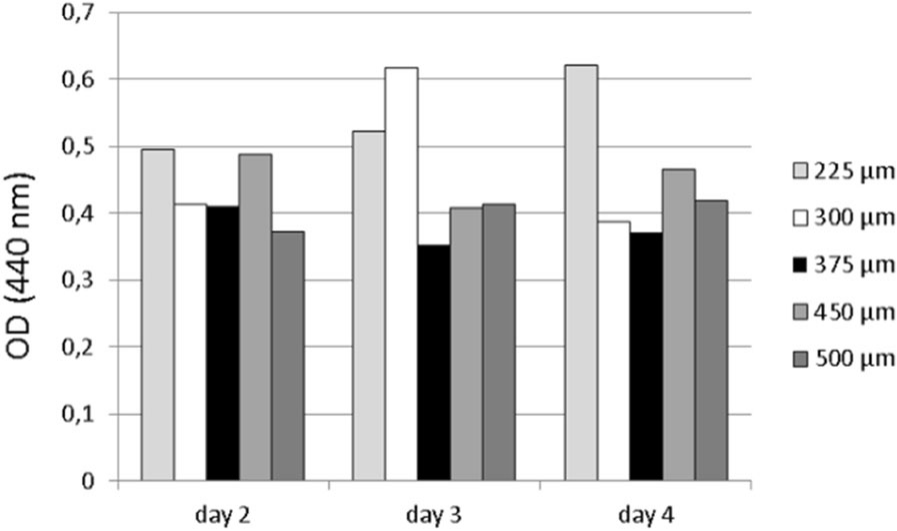
Cell viability displayed in Fig. 3 in direct comparison, itemized by box size

WST-1 testing revealed good cell proliferation of MG63 cells on all PCL scaffolds (Fig. 3). Cell proliferation on all scaffolds of all box sizes always remained more- or less distinct behind the control groups consisting of MG63 cells growing on cell culture plates (polystyrene). For the 225 μm and 500 μm box-size scaffolds, a progression of cell viability could be observed over time. The other sizes revealed inconsistent growth behavior (Fig. 4). Overall, 225 μm scaffolds displayed the highest viability, with a general decrease towards the larger box sizes.

**Fig. 5:**
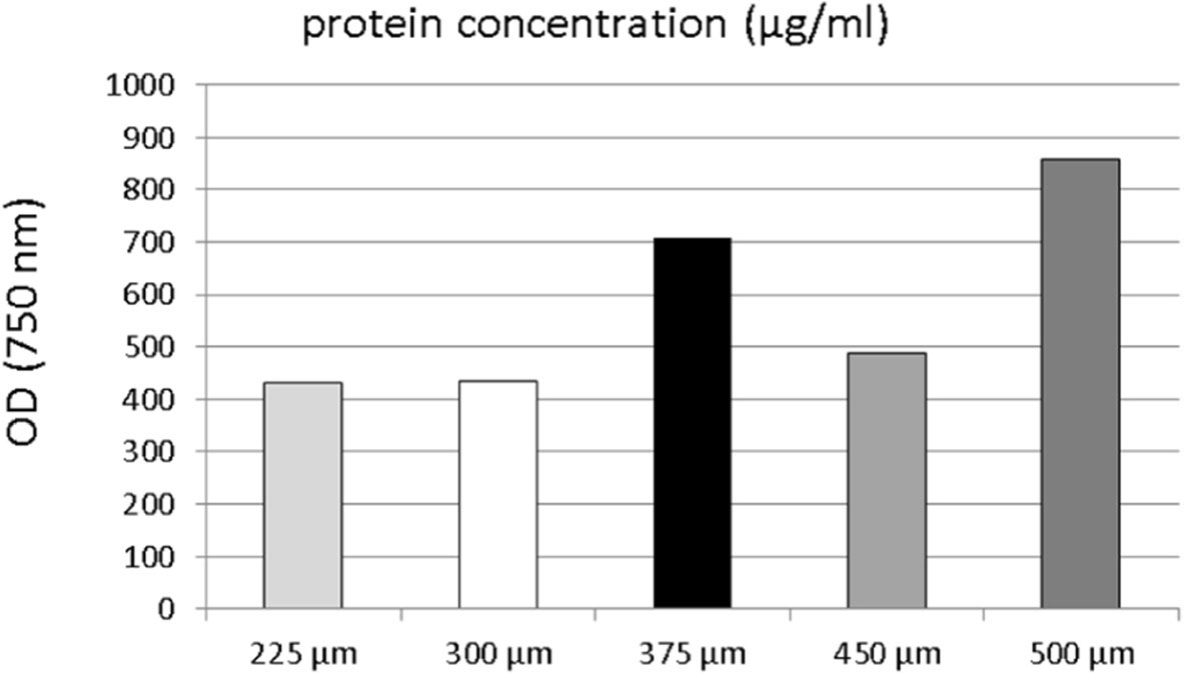
Protein concentration in MG63 cells seeded on MEW box-structured PCL scaffolds of different box-sizes according to Lowry method testing after 4 days of cell culture

The measurement of the protein concentration of MG63 cells seeded on differently sized scaffolds yielded values between 432.5 μg/ml and 858.5 μg/ml. Substantial differences could generally not be found (Fig. 5). Only 500 μm scaffolds displayed higher protein concentrations than the 225 μm and 300 μm scaffolds.

**Fig. 6:**
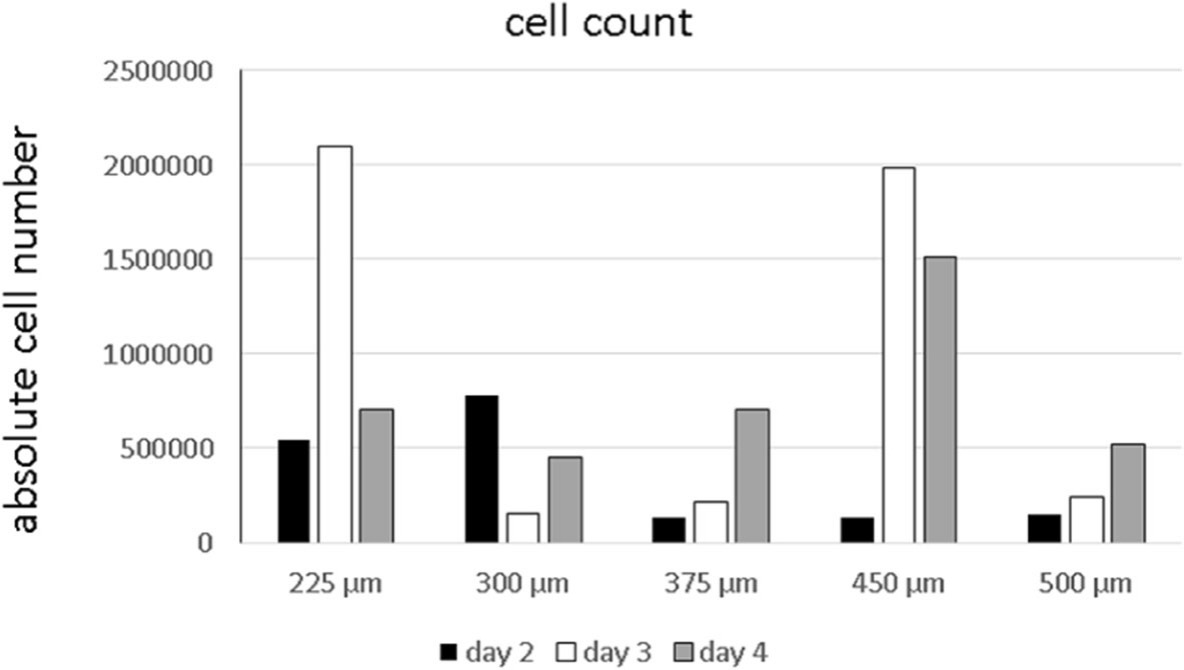
Cell count of MG63 cells seeded on MEW box structured PCL scaffolds of different box-sizes after 2, 3 and 4 days of cell culture

Cell counting revealed the presence of viable MG63 cells over the 4 days (Fig. 6). The cell counts varied between 21.0 × 10^5^ (225 μm, day 3) and 1.3 × 10^5^ (450 μm, day 2). Most viable MG63 cells were found on scaffolds of 225 μm and 450 μm box sizes. A uniform trend could not be found.

**Fig. 7:**
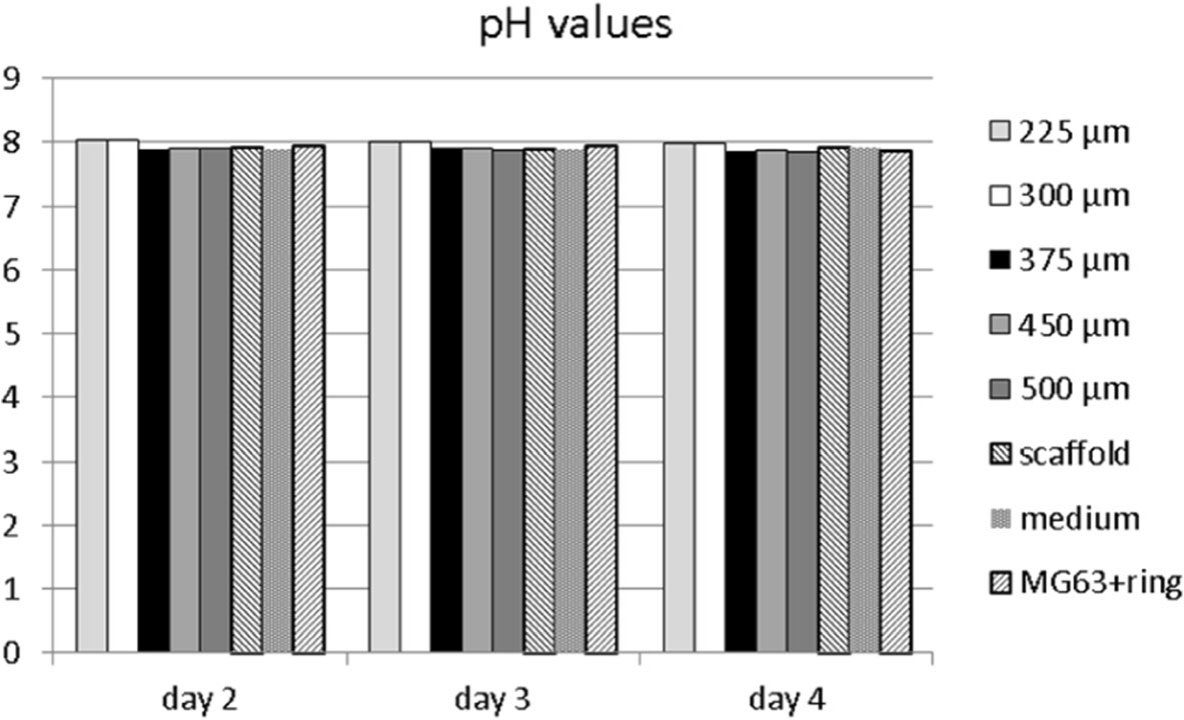
pH values in cell culture medium of MG63 cells seeded on MEW box-structured PCL scaffolds of different box-sizes after 2, 3, and 4 days of cell culture. Scaffold: MEW PCL scaffolds burdened with glass ring/beads, Medium: DMEM cell culture medium, MG63 + ring: MG63 cells settled upon polystyrene cavities of multiwell plates in presence of glass ring/beads

An analysis of the pH values of the cell culture supernatant revealed physiological values (Fig. 7) that consistently lay within the range of 7.85 and 8.03 and complied with values of the pure cell culture medium.

### Morphological characterization

**Fig. 8:**
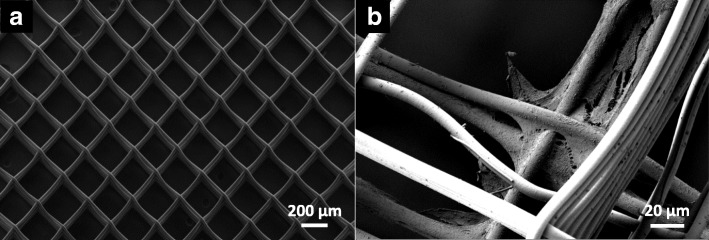
Exemplary SEM micrograph of MEW PCL-scaffold. **a**) Overview. Box size is 225 μm and box structures are built from 2 × 5 layers of PCL fibers. **b**) MG63 cells after 4 days of cell culture

Figure 8 displays an example of a MEW PCL scaffold with a box size of 250 μm. Exact fiber placement and box geometry can be seen. SEM micrographs further exhibited growth of viable MG63 cells with flattened morphology and cell protrusions mostly at the intersections of polymer fibers of different layers.

**Fig. 9:**
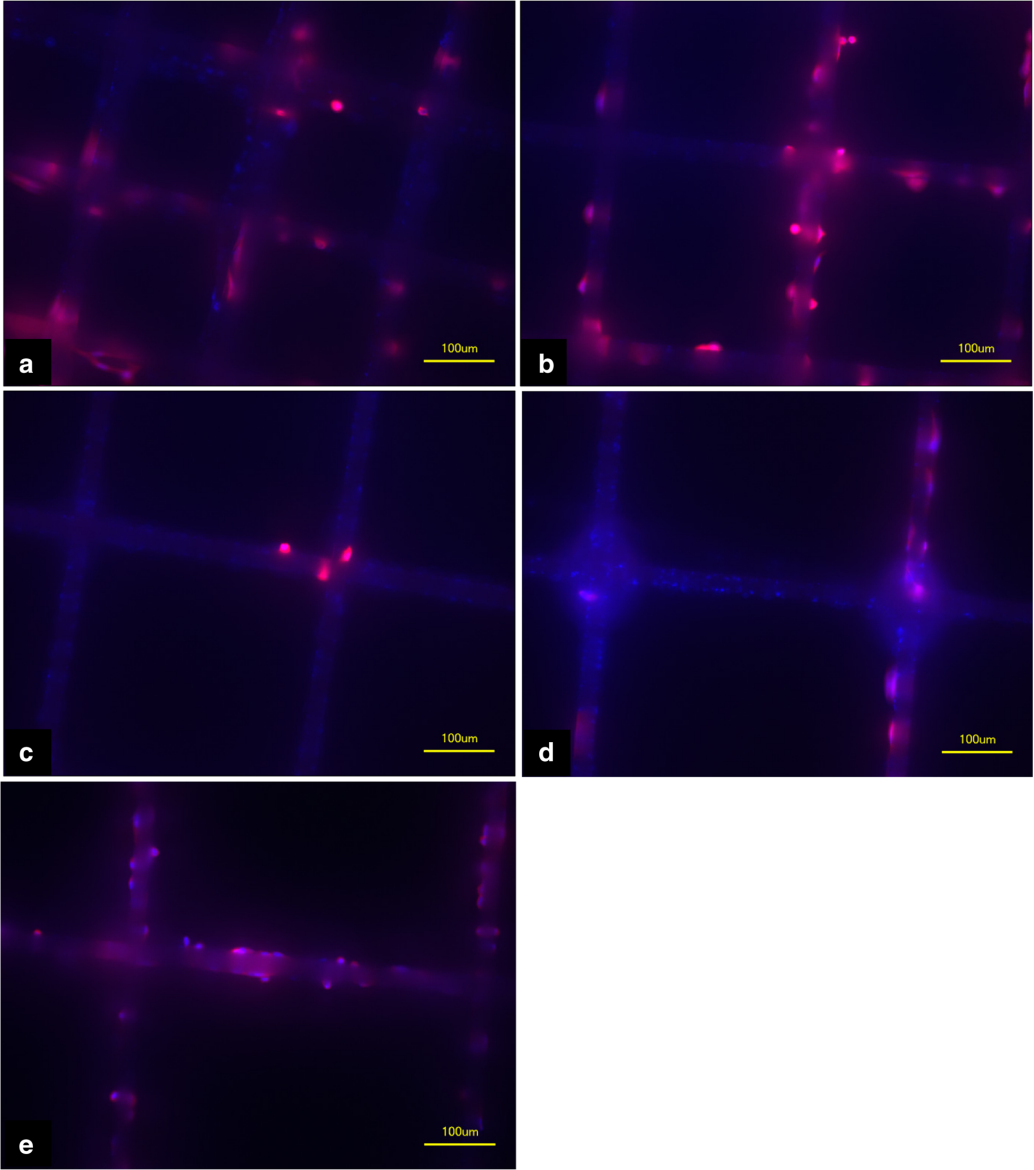
Phalloidin staining of MEW PCL-scaffolds after 4 days of cell culture with MG63 cells. **a**) Box size 225 μm, **b**) box size 300 μm, **c**) box size 375 μm, **d**) box size 450 μm, **e**) box size 500 μm

**Fig. 10:**
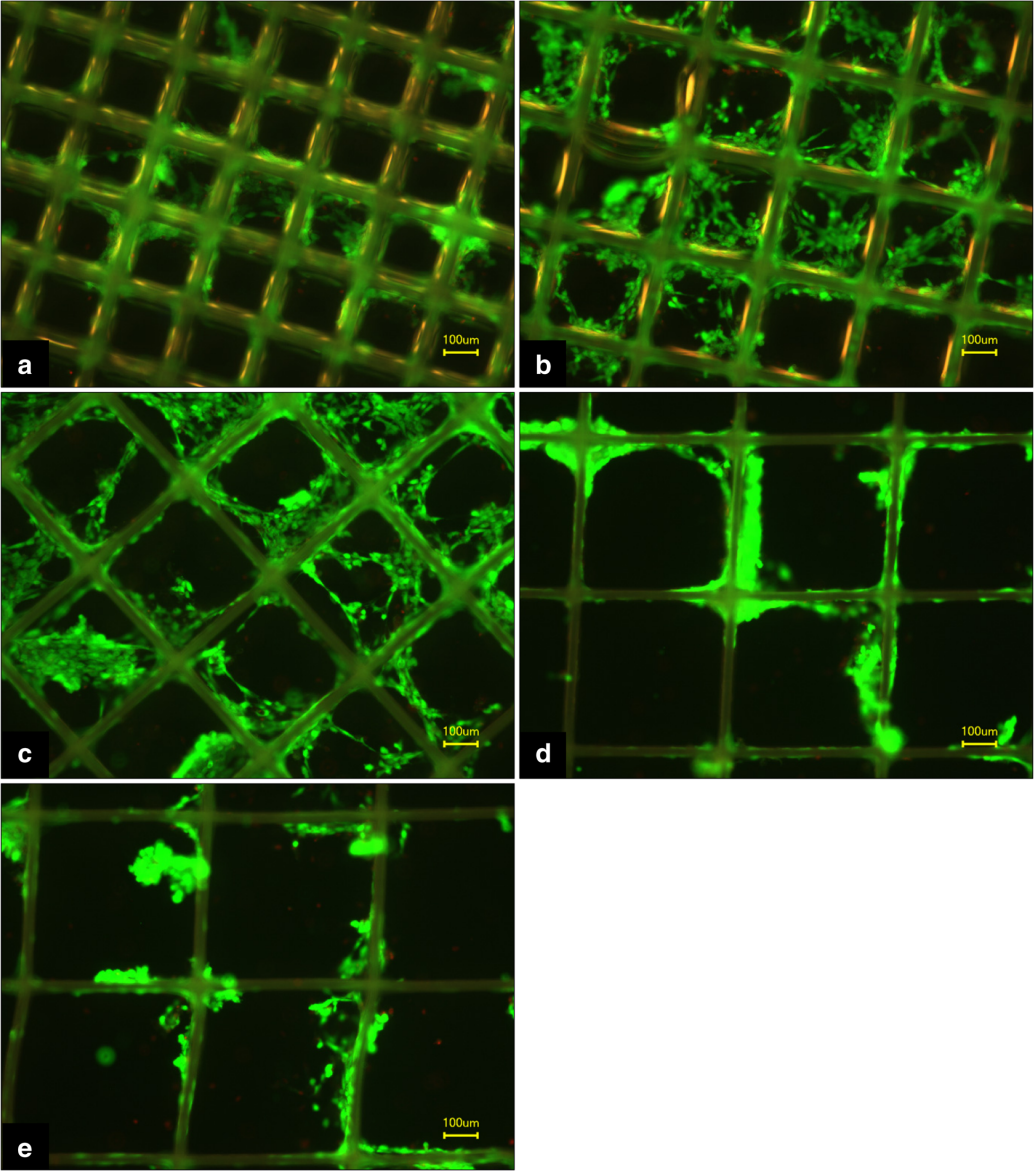
FDA/PI staining of MEW PCL-scaffolds after 4 days of cell culture with MG63 cells. Green staining displays living cells; red staining, dead cells; **a**) Box size 225 μm, **b**) box size 300 μm, **c**) box size 375 μm, **d**) box size 450 μm, **e**) box size 500 μm

Histopathological FDA/PI and phalloidin staining also revealed good cell attachment (Figs. 9 and 10) for the different box sizes. Viable cells could be found not only at intersections but also along straight fibers.

## Discussion

The aim of this pilot study was to develop scaffolds for an application in oral and maxillofacial surgery to overcome the drawbacks of currently used membrane systems. To achieve this goal, we used MEW as an emerging technology in tissue engineering that enables the fabrication of scaffolds in almost unlimited designs without the use of toxic components. The long-term objective is to generate multilayered scaffolds for oral regeneration that simultaneously fulfill all requirements for a sufficient regeneration of oral tissues, such as mucosa and bone. These multilayered membranes should fulfill all requirements of GBR such as complete cell occlusivity, which is not yet realized with the scaffolds used in our study, as they have relatively wide fiber spacings up to 500 μm. Nevertheless, as a first step towards this goal, in this pilot study, we examined the possibility of fabricating MEW scaffolds for bone regeneration and of influencing the growth behavior of osteoblast-like cells through a variation of scaffold designs.

Thus far, various studies have dealt with the use of electrospun scaffolds in tissue engineering of different tissues, such as vascular tissue, neural tissue, and bone tissue [17–21]. Most of them have indicated that MEW is a favorable method of fabricating scaffolds for tissue regeneration. However, only a few studies have investigated the use of MEW scaffolds and membranes as a means of enhancing oral wound healing [22, 23]. As good cytocompatibility is a basic requirement for the application of scaffolds and membranes in humans, this study investigated the cell growth of typical cells of oral soft- and hard tissues upon MEW scaffolds. PCL has already proven its role as a valuable tissue engineering material [24–26]. A good biodegradability as well as an excellent biocompatibility are key features in this. Besides, the relatively low melting point of approximately 63 °C makes PCL is very suitable for the MEW process [4, 27]. Especially in bone regeneration, PCL scaffolds yielded good results considering cell viability and cell proliferation [21, 28]. Only medical-grade PCL was used for the polymer melt for scaffold fabrication in this study so that a translation to the clinical routine would basically be possible. In this manner, high-precision and well-defined box-shaped scaffolds were produced without the use of toxic organic solvents or any other agents.

The architecture of scaffolds, size, morphology and interconnectivity play an important role in the sufficient regeneration of bone [29]. Various studies have dealt with the issue of an optimal pore size for scaffolds in bone-tissue engineering thus far [30–33]. The range of pore sizes regarded as ideal here varies between 95 μm and 500 μm; however, these values refer neither to electrospun scaffolds nor to PCL as the basic material that we examined in this study. To bridge this gap, the sizes of the box structures were varied within the range, that is described in current literature (225 μm, 300 μm, 375 μm, 450 μm and 500 μm scaffolds), to see if this would lead to better cell growth of osteoblast-like MG63 cells on the medical-grade PCL scaffolds. Cell growth was then evaluated by means of WST-1 testing, protein-concentration measurement, and cell counting at different time points. A slightly better cell viability was generally observed for smaller box sizes, but a definite trend, which of the examined box-sizes between 225 μm and 500 μm promoted primary cell attachment of osteoblast-like cells best, could not be found. Better cell viability for smaller box sizes can be explained by the higher surface area for cell adhesion. Although all scaffolds had the same dimensions, smaller box sizes contain more fibers due to the tighter arrangement of these fibers. In general, decreased cell growth on all scaffolds compared with the positive control groups can be partly attributed to the experimental setup. MG63 cells display a spherical morphology before seeding on scaffolds so that initial seeding on the thin fibers is hard to achieve; most of the cells might primarily fall through the meshes. Cell settlement might have occurred in large parts through cells that had primarily been on the bottom of the well and then migrated onto the scaffold.

Considering protein concentration, all examined scaffolds with box sizes between 225 μm and 500 μm yielded similar results. A considerable impact of different box sizes towards the protein status of MG63 cells was not observed. A similar situation was found regarding the cell counting of adherent cells. Although viable MG63 cells could be detected at all times during the experiment on the scaffolds, different box sizes did not seem to have a significant influence on either cell adhesion or the growth behavior of the osteoblast-like cells.

To roughly assess the potential influence of PCL scaffolds on the surrounding milieu in terms of alkaline or acidic changes, the pH values of cell culture supernatant were observed on each day of the experiment. As a degradation of the scaffolds at this early stage was not probable, at no point non-physiological values were found, which indicates - as expected - that within the first few days, PCL showed no negative influence in terms of acidic or alkaline milieu changes that may impair wound-healing process [34].

The morphological analysis of MEW scaffolds revealed an accurate layer-by-layer deposition of PCL fibers, which formed the desired pre-defined geometry of interconnecting boxes. The accuracy of MEW in creating complex porous structures has been previously described. Filament diameters are in a low submicron range, but sufficiently large interconnecting pores for cell growth can be found throughout the scaffolds [6, 9]. In our study cell growth upon scaffolds was observed in the initial stage of the cell culture mostly at fiber intersections in SEM micrographs, while staining also revealed cell growth alongside straight fiber parts.

Using MEW with medical-grade polymers to produce scaffolds for improved regeneration of oral tissues provides an opportunity to generate individually planned, high-precision, non-toxic biomaterials that can basically be directly used in humans. Thanks to MEW’s precise printing capabilities, the geometry of the scaffolds can largely be chosen at will. This way, individual designs for individual cells types/tissues can be realized. PCL scaffolds with pore sizes optimized for osteoblast growth may significantly promote bone healing. Furthermore, stratified scaffolds that contain different MEW layers also enable enhanced wound healing for oral mucosa and bone. By fabricating scaffolds that allow proper osteoblast attachment, we made the first move towards this goal. Further steps such as optimizing scaffold surfaces for better cell adhesion and fabricating multilayered MEW scaffolds tailored to different tissue types, need to follow.

Such scaffolds/membranes would prove beneficial, especially in the field of oral and maxillofacial surgery, where impaired wound healing in the alveolus region oftentimes poses problems. Furthermore, a pre-implantological use of these membranes seems suitable. In atrophic regions of the alveolus in which elaborate bone augmentation is necessary, coverage with membranes that additionally promote bone healing should prove a promising approach to improved GBR.

## Conclusions

Using MEW, we were able to produce scaffolds out of medical-grade polymer PCL, which enabled good cell attachment for osteoblasts. SEM imaging revealed accuracy for the MEW process with exactly arranged fibers with a diameter of 20 μm. Scaffolds with box geometries of different sizes between 225 μm and 500 μm were examined. In this range, a preferred box size for initial osteoblast attachment could not be found according to cell viability, cell count, or protein concentration of cells. Nevertheless, all box sizes proved to be a good substrate for osteoblast cell growth. To further enhance osteoblast attachment, surface modifications, such as coating the fibers with calcium phosphate, seem favorable. Overall, further laboratory testing and subsequent clinical trials are required in order to enhance membrane properties and transfer them into clinical routine.

## Abbreviations

3D: Three dimensional
CAD: Computer-aided design
CAM: Computer-aided manufacturing
DMEM: Dulbecco’s modified eagle’s medium
FCS: Fetal calf serum
FDA/PI: Fluorescindiacetate/Propidiumiodide
GBR: Guided bone regeneration
GTR: Guided tissue regeneration
MEW: Melt electrospinning writing
MRONJ: Medication-related osteonecrosis of the jaw
P/S: Penicillin and streptomycin
PBS: Phosphate buffered saline
PCL: Polycaprolactone
SEM: Scanning electron microscopy
WST: Water-soluble tetrazolium
